# Association of Mu-Opioid Receptor(MOR) Expression and Opioids Requirement With Survival in Patients With Stage I-III Pancreatic Ductal Adenocarcinoma

**DOI:** 10.3389/fonc.2021.686877

**Published:** 2021-06-18

**Authors:** Hao Zhang, Mengdi Qu, Aysegul Gorur, Zhirong Sun, Juan P. Cata, Wankun Chen, Changhong Miao

**Affiliations:** ^1^ Department of Anaesthesiology, Zhongshan Hospital, Fudan University, Shanghai, China; ^2^ Department of Anaesthesiology and Perioperative Medicine, The University of Texas MD Anderson Cancer Centre, Houston, TX, United States; ^3^ Anaesthesiology and Surgical Oncology Research Group, Houston, TX, United States; ^4^ Department of Anaesthesiology, Fudan University Shanghai Cancer Centre, Shanghai, China; ^5^ Department of Oncology, Shanghai Medical College, Fudan University, Shanghai, China; ^6^ Fudan Zhangjiang Institute, Shanghai, China

**Keywords:** mu-opioid receptor, OPRM1, opioids, pancreatic cancer, overall survival

## Abstract

**Background:**

The use of opioids in patients with metastatic pancreatic ductal adenocarcinoma (PDAC) is associated with shorter survival and not dependent on the expression of the mu-opioid receptor (MOR). The role of opioid use and MOR expression in stage I-III PDAC has not been investigated.

**Methods:**

We conducted retrospective study in patients with stage I-III PDAC. MOR expression and *OPRM1* gene expression in tumour tissue and non-tumour tissue was measured. Primary endpoints were overall survival (OS) and disease-free survival (DFS). Secondary endpoints included perineural invasion, intraoperative sufentanil consumption, and length of stay. We performed a subgroup group analysis to evaluate the interaction between levels of MOR expression, amount of opioids use (high *versus* low) and its association with survival.

**Results:**

A total of 236 patients were enrolled in this study.There were no significantly difference in OS rates in patients with high *versus* low levels of MOR (1-year OS: 65.2% *versus* 70.6%, P=0.064; 3-year: 31.4% *versus* 35.8%, P=0.071; 5-year: 19.4% *versus*. 16.2%, P=0.153, respectively) in the tumours. The DFS rates between the groups were no significantly difference. Of note, a high expression of MOR combined with high opioid consumption was associated with poor prognosis in stage I-III PDAC patients. Tumor expressing high levels of MOR show higher rates of perineural invasion.

**Conclusion:**

MOR is not an independent predictor of poor survival in stage I-III PDAC but associated with perineural invasion. Patients requiring high amounts of opioids intraoperatively show worse outcome if they are expressing high levels of MOR.

## Introduction

Pancreatic ductal adenocarcinoma (PDAC) is one of the deadliest cancers worldwide (1). Owing to the lack of appropriate methods for early detection, the five years survival rate for PDAC is only 8% (2). This worrisome statistic highlights the need of identifying actionable tumour targets to achieve better oncologic control of the disease and thus prolong the survival of patients with PDAC.

In patients with early stage PDAC, surgery is still the treatment of choice (3).

Opioids are still the main analgesics administered to provide adequate pain control during and after PDAC surgery (4). Opioids act on the mu-opioid receptor (MOR) to produce analgesia; however, the receptor is also located in cancer cells such as PDAC cells (5). Over the past few years, there has been increasing interest to elucidate whether MOR or its encoding gene *OPRM1* can be used as predictive biomarkers in different cancers (5). Studies indicate that a high expression of MOR in malignant specimens were associated with worse survival outcome in patients with lung cancer, hepatocellular carcinoma, and laryngeal squamous cell carcinoma (6–8). However, other studies have found that high levels of expression of MOR were not associated with poor prognosis in colorectal and oesophageal squamous cell carcinomas (9, 10). The prognosis significance of MOR expression in PDAC has been recently investigated in patients with advanced disease (11). While a retrospective study demonstrated that the expression of MOR did not impact the prognosis, a high opioid consumption was associated with decreased survival (11).

The association of MOR expression and long-term outcomes in patients with non-metastatic pancreatic cancer is still unclear. We conducted a retrospective study to assess the association between MOR expression levels and long-term outcomes in non-metastatic PDAC patients. We hypothesized that MOR expression is increased in pancreatic cancers in comparison to normal pancreatic tissue and is associated with shorter long-term survival. Furthermore, we investigated the association between MOR expression and perineural invasion, intraoperative opioids consumption and hospital stay.

## Materials and Methods

### Study Population

We conducted retrospective analysis after obtaining approval from Fudan University Shanghai Cancer center (Protocol#2020106-1). We included patients scheduled for pancreatectomy from January 2015 to December 2017. All patients included in the study signed an informed consent after being admitted to the hospital. Inclusion criteria included as follows: (a) surgery for stage I-III PDAC; (b) R0 resection for confirmed PDAC; and (c) no history of another malignant tumour. We excluded patients who died within 30 days of surgery complications and those without complete clinicopathological and follow-up data.

### Measurements and Outcomes

The primary outcomes of this research were overall survival (OS) and disease-free survival (DFS). OS was defined as the period from the end of the pancreatic surgery to death or the last follow-up date. DFS was defined as the period from the date of surgery to the date of tumour recurrence or December 2019. Secondary outcomes included perineural invasion, intraoperative sufentanil consumption, and duration of hospital length of stay (LOS).

### Anaesthesia Care

In the operating room, patients were routinely monitored according to American Society of Anaesthesiologists (ASA) standards. Induction of general anaesthesia was performed with target-controlled infusion propofol (3.0-4.0μg/ml), sufentanil (0.3-0.5μg/kg), and rocuronium (0.5mg/kg). After induction of general anaesthesia, patients were intubated and anaesthesia was maintained with 2.0-3.0% sevoflurane in mixture oxygen/air. Sufentanil and rocuronium were given intravenously during the surgery according to clinical judgment.

### Immunohistochemistry

PDAC tissue fixed on paraffin blocks were obtained for immunohistochemistry (IHC). IHC staining was performed as previously described (8). Briefly, the anti-Mu Opioid Receptor (UMB3)-C-terminal (ab134054) antibody was used in a concentration of 1:200. Secondary antibodies anti-Goat Anti-Rabbit IgG H&L (HRP) (ab205718) were used. Two investigators (physician pathologists) blinded to the data were asked to assessed and scored MOR expression based on previously published criteria (8). The total MOR score was calculated based on staining intensity and proportion of immun0possitive in cancer cells (10).

### Impact of OPRM1 on Survival

In addition, we investigated whether mRNA levels of the gene coding for MOR (*OPRM1*) in PDAC were associated with changes in DFS and OS. Briefly, the association of *OPRM1* mRNA expression levels on OS and DFS was assessed *via* Kaplan-Meier Plotter (https://kmplot.com/analysis/). Kaplan-Meier Plotter is a public database containing multiple microarray datasets including GEO, EGA and TCGA (12). The analyses were run on 177 PDAC patients. We included 69 and 146 patients in OS and RFS analyses respectively.

### Statistical Analysis

Continuous variables were analysed as mean and standard deviation (SD) or median and interquartile. Frequency counts and percentages were calculated for categorical variables. Univariate associations between MOR expression and clinical variables were tested with Chi-square test. OS and DFS analyses were assessed by Kaplan-Meier methods. Hazard ratios (HR) with corresponding 95% confidence intervals (CI) were calculated. Univariate Cox proportional hazards models were fitted to evaluate the effects of continuous variables on the time-to-event outcomes. Multivariable Cox proportional hazard models were used including important and significant covariates. In order to reduce bias during selection, a propensity score matching analysis was performed to compare OS and DFS between patients who have high *versus* low levels of MOR expression. Patients were matched using a 5-to-1 digit Greedy match algorithm. Eight variables were used in the model including age, ASA physical status, tumour differentiation, Charlson comorbidity index (CCI), Tumor Nodes Metastasis (TNM) stage, surgery type, tumour location and administration of adjuvant chemotherapy. Mean cut-off values for MOR expression and intraoperative opioids consumption were used for subgroup survival analysis using X-tile software (13). For *OPRM1* analysis, the Log-rank test with 95% confidence interval and p values were automatically calculated by the Kaplan-Meier Plotter software. Statistical analyses were performed with SPSS version 17.0. A P value < 0.05 was considered statistically significant.

## Results

Four-hundred and twenty-seven patients were screened for this study. After meet exclusion criteria, 146 patients were excluded because of concomitant cancers (n=27); distant metastasis (n=69); chronic inflammatory diseases (n=26) and missing follow-up data (n=24). Two-hundred eighty-one patients were finally included in analysis (**Supplementary Figure 1A**). Baseline demographic and tumour characteristics are shown in **Table 1**. MOR expression levels were not significantly associated with age (P=0.726), gender (P=0.893), CCI (P=0.946), tumour differentiation (P=0.727), tumour size (P=0.757), TNM stage (P=0.766) and tumour location (P=0.446) (**Table 1**).

**Table 1:** Baseline characteristics of patients in both groups.

Variable	Original cohort		*P*	Matched cohort		*P*	Standard difference
	MOR high expression group (n=145)	MOR low expression group (n=136)		MOR high expression group (n=118)	MOR low expression group (n=118)		
**Age (media-IQR, year)**	56 (45-63)	55 (46-67)	0.726	56 (46-62)	56 (46-66)	0.684	3.15
**Gender (n, %)**							
Female	48 (33.2%)	44 (32.0%)	0.893	42 (35.6%)	43 (36.4%)	0.892	–
Male	97 (66.8%)	92 (68.0%)		76 (64.4%)	75 (63.6%)		–
**BMI kg/m2, (median-IQR)**	23.5 (21.3-24.3)	22.6 (20.8-24.8)		22.3 (21.3-23.9)	23.0 (20.7-24.3)		–
**ASA (n, %)**			0.881			0.948	2.58
I	94 (65.2%)	89 (65.4%)		76 (64.4%)	74 (62.7%)		–
II	38 (26.4%)	37 (27.2%)		35 (29.6%)	36 (30.5%)		–
III	13 (8.4%)	10 (7.4%)		7 (6%)	8 (6.8%)		–
**Patients enrolled**			0.969			0.915	–
2015	50 (34.5%)	46 (33.6%)		39 (33.1%)	41 (34.7%)		
2016	47 (32.4%)	46 (33.8%)		38 (32.2%)	39 (33.1%)		
2017	48 (33.1%)	44 (32.6%)		41 (34.7%)	38 (32.2%)		
**CCI (n, %)**			0.946			0.861	2.89
0	91 (62.8%)	87 (63.9%)		79 (66.9%)	75 (63.5%)		–
1	43 (29.7%)	38 (27.8%)		30 (25.4%)	33 (27.9%)		
≧2	11 (7.5%)	11 (8.3%)		9 (7.7%)	10 (8.9%)		
**Tumor differentiation (n, %)**			0.727			0.893	3.26
Well-moderate	53 (36.8%)	47 (34.8%)		43 (36.4%)	44 (37.3%)		–
Poor	92 (63.2%)	89 (65.2%)		75 (63.6%)	74 (62.7%)		–
**T stage (n, %)**			0.542			0.404	
1	35 (23.9%)	31 (22.8%)		30 (25.4%)	29 (24.6%)		–
2	95 (65.7%)	85 (62.6%)		80 (67.8%)	75 (63.6%)		–
3	15 (10.4%)	20 (14.6%)		8 (6.8%)	14 (11.8%)		–
**N stage (n, %)**			0.914			0.836	
0	60 (41.2%)	55 (40.5%)		54 (45.7%)	52 (44.1%)		–
1	56 (38.7%)	51 (37.6%)		51 (43.2%)	50 (42.4%)		–
2	29 (20.1%)	30 (21.9%)		13 (11.1%)	16 (13.5%)		–
**AJCC 8^th^ edition TNM stage (n, %)**			0.766			0.392	4.15
I	70 (48.8%)	63 (46.5%)		54 (45.7%)	52 (44.1%)		–
II	56 (38.7%)	51 (37.9%)		52 (44.1%)	47 (39.8%)		–
III	19 (12.5%)	22 (15.6%)		12 (10.2%)	19 (16.1%)		–
**Tumor size (n, %)**			0.757			0.613	3.69
≤2 cm	110 (75.8%)	101 (74.2%)		98 (83.1%)	95 (80.5%)		–
>2 cm	35 (24.2%)	35 (25.8%)		20 (16.9%)	23 (19.5%)		–
**Surgery type (n, %)**			0.660			0.628	3.54
Pancreaticoduodenectomy	82 (56.7%)	74 (54.7%)		71 (60.2%)	68 (57.6%)		–
Distal pancreatectomy	46 (31.4%)	41 (30.0%)		34 (28.8%)	32 (27.1%)		–
Total pancreatectomy	17 (11.9%)	21 (15.3%)		13 (11.0%)	18 (15.3%)		–
**Tumor location (n, %)**			0.446			0.777	4.56
Head of pancreas	99 (68.5%)	87 (64.2%)		83 (70.3%)	81 (68.6%)		
Tail of pancreas	46 (31.5%)	49 (35.8%)		35 (29.7%)	37 (31.4%)		
**Estimated blood loss (n, %)**			0.739			0.757	–
≤ 400 ml	106 (73.5%)	97 (71.6%)		92 (77.9%)	90 (76.3%)		–
> 400 ml	39 (26.5%)	39 (28.4%)		26 (22.1%)	28 (23.7%)		–
**Blood transfusion**			0.858			0.678	–
No	131(90.5%)	122 (89.7%)		104 (88.1%)	106 (89.8%)		
Yes	14 (9.5%)	14 (10.3%)		14 (11.9%)	12 (10.2%)		
**Postoperative chemotherapy (n, %)**			0.689			0.619	5.21
Yes	122 (84.4%)	112 (82.6%)		97 (82.2%)	94 (79.6%)		–
no	23 (15.6%)	24 (17.4%)		21 (17.8%)	24 (20.4%)		–

BMI, Body Mass Index; IQR, Inter Quartile Range; ASA, American Society of Anesthesiologists score; CCI, Charlson Comorbidity Index; AJCC 8th TNM stage, American Joint Committee on Cancer the 8th edition; MOR, Mu-Opioids Receptor.

**Figure 1:**
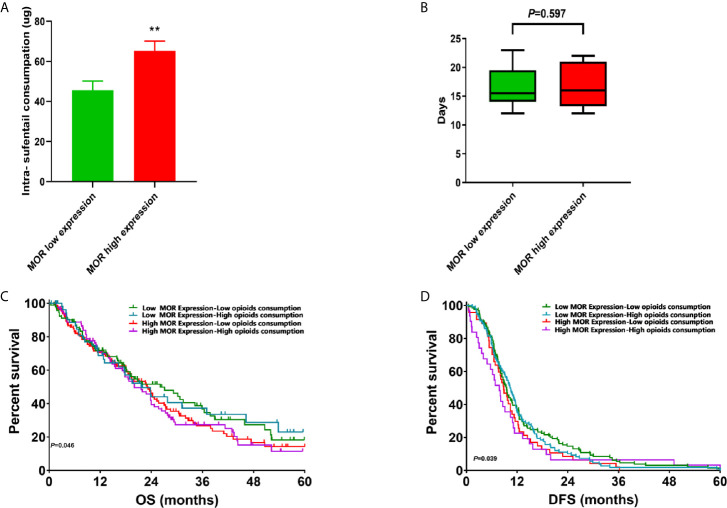
**(A)** Intraoperative sufentanil consumption according to MOR expression; **(B)** Length of stay according to MOR expression; **(C)** Subgroup analysis of OS curves according to MOR expression and opioids consumption. **(D)** Subgroup analysis of DFS curves according to MOR expression and opioids consumption. **P < 0.05.

**Figure 2:**
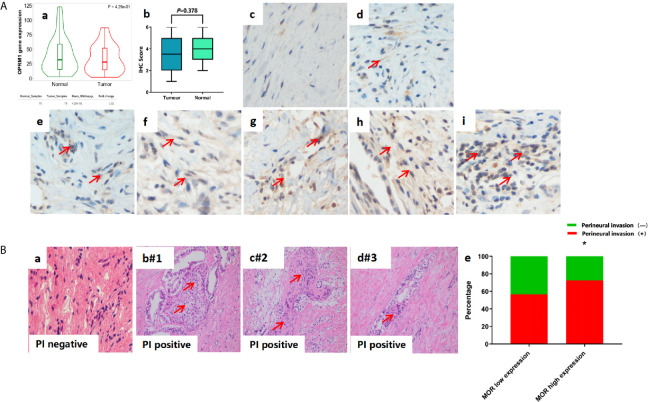
**(A)** Representative image of IHC sample to describe scoring and MOR expression. All images are magnitude 400. (a) OPRM1 gene expression in PDAC tumour tissue and adjacent non-tumour tissue; (b) MOR expression in PDAC tumour tissue and adjacent non-tumour tissue; (c) score 0; (d) score 1; (e) score 2; (f) score 3; (g) score 4; (h) score 5; (i) score 6. **(B)** Representative image of HE staining sample to describe Perineural invasion (PI). (a) PI negative. (b-d) PI positive patients (#1-3). (e) PI positive according to MOR expression. *P < 0.001.

Immunohistochemistry studies showed no significant differences between MOR expression levels in the tumour and adjacent non-tumour tissues (P=0.378, **Figure 1A**). Similarly, there were no significantly differences between normal and tumour tissue in *OPRM1* expression levels (P=0.429, **Figure 2A**).

### Primary Outcomes

**Figure 3:**
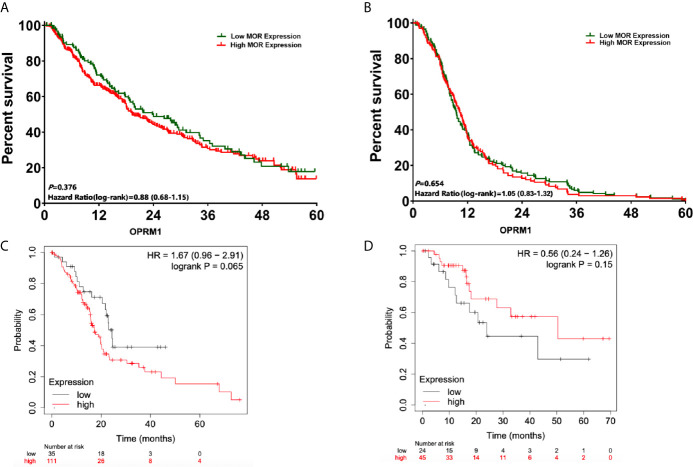
Survival analysis from the date of pancreatic cancer surgery according to expression of MOR and OPRM1. **(A)** OS analysis according to MOR expression; **(B)** DFS analysis according to MOR expression; **(C)** OS curves according to expression of OPRM1 from the cancer database; **(D)** DFS curves according to expression of OPRM1 from the cancer database.

The median follow-up time for patients included in the analysis was 15.8 months (95%CI, 13.4, 16.7). After propensity score matching, there were no significantly difference in 1-, 3- and 5-year OS between patients with high *versus* low levels of expression MOR (1-year OS: 65.2% *versus* 70.6%, P=0.064; 3-year: 31.4% *versus* 35.8%, P=0.071; 5-year: 19.4% *versus* 16.2%, P=0.153, respectively, **Figure 3A**). The univariate Cox regression analysis showed that poor tumour differentiation (P<0.001), perineural invasion (P<0.001), and lack of adjuvant chemotherapy (P<0.001) were associated with worse OS (**Table 2**). Also, poor tumour differentiation (HR: 1.85, 95%CI: 1.06, 2.32, P=0.019), nerve invasion positive (HR: 1.58, 95%CI: 1.13, 1.61, P=0.042), and no postoperative chemotherapy (HR: 1.64, 95%CI: 1.02, 1.78, P<0.001) were independent predictors of reduced OS after adjusting for clinical and histopathological factors (**Table 3**). The association between high MOR expression and OS was not statistically significant in the model (HR: 1.08, 95%CI: 0.96, 1.38, P=0.125, **Table 3**). The *OPRM1* gene expression level did not significantly affect OS (P=0.065, **Figure 3C**).

**Table 2:** Univariate analysis of OS and DFS.

Variables	OS		DFS	
	HR (95% CI)	*P*-value	HR (95% CI)	*P*-value
**Age**	1.02 (0.98,1.21)	0.258	1.08 (0.88,1.32)	0.267
**Gender (Male *vs.* Female)**	1.11 (0.72,1.52)	0.324	1.05 (0.94,1.11)	0.451
**ASA score (1 *vs.* 2. *vs.* 3)**	1.05 (0.70,1.46)	0.159	1.11 (0.98,1.16)	0.298
**CCI (0 *vs.*1 *vs.* ≧2)**	1.15 (0.64,1.20)	0.267	1.26 (1.08,1.61)	0.187
**Tumour differentiation (Poor)**	2.10 (1.71,2.76)	<0.001	2.34 (1.58,2.65)	<0.001
**Nerve invasion (Yes)**	1.52 (1.24,1.75)	<0.001	1.63 (1.35,1.81)	<0.001
**AJCC TNM stage (I *vs.* II *vs.* III)**	1.45 (0.93,1.71)	0.254	1.34 (0.98,1.71)	0.186
**Postoperative Chemotherapy (No)**	1.42 (1.29,1.58)	<0.001	1.64 (1.38,1.81)	<0.001
**MOR expression (high)**	1.12 (0.94,1.46)	0.078	1.18 (0.97,1.24)	0.175

BMI, Body Mass Index; IQR, Inter Quartile Range; ASA, American Society of Anaesthesiologists score; CCI, Charlson Comorbidity Index; AJCC 8th TNM stage, American Joint Committee on Cancer the 8th edition; OS, Overall Survival; DFS, Disease free Survival; MOR, Mu-Opioids Receptor.

**Table 3:** Multivariable Cox proportional of OS and DFS.

Variables	OS (Before matching)		OS (After matching)		DFS (Before matching)		DFS (After matching)	
	HR (95% CI)	*P*-value	HR (95% CI)	*P*-value	HR (95% CI)	*P*-value	HR (95% CI)	*P*-value
**Tumour differentiation (Poor)**	1.95 (1.10,2.40)	0.034	1.85 (1.06,2.32)	0.019	2.45 (1.02,2.60)	0.034	2.02 (1.02,2.42)	0.027
**Nerve invasion (Yes)**	1.64 (1.13,1.76)	0.012	1.58 (1.13,1.61)	0.042	1.89 (1.06,1.96)	0.019	1.75 (1.10,1.86)	0.032
**Postoperative Chemotherapy (No)**	1.54 (1.01,1.98)	<0.001	1.64 (1.02,1.78)	<0.001	1.63 (1.22,1.79)	<0.001	1.54 (1.32,1.70)	<0.001
**MOR expression (high)**	1.25 (0.93,1.70)	0.264	1.08 (0.96,1.38)	0.125	1.23 (0.90,1.72)	0.326	1.19 (0.94,1.43)	0.167

OS, Overall Survival; DFS, Disease free Survival; MOR, Mu-Opioids Receptor.

Similarly, there were no significantly difference in DFS when comparing MOR high *versus* MOR low expression levels (1-year DFS: 40.6% *versus* 38.9%, P=0.248; 3-year: 8.6% *versus* 8.1%, P=0.657; 5-year: 2.5% *versus* 2.2%, P=0.843, respectively, **Figure 3B**). The univariate Cox regression analysis showed that patients with poor tumour differentiation (P<0.001), perineural invasion (P<0.001), and those not receiving adjuvant chemotherapy (P<0.001) had a reduced DFS (**Table 2**). In the multivariate analysis, poor tumour differentiation (HR: 2.02, 95%CI: 1.02, 2.42, P=0.027), perineural invasion (HR: 1.75, 95%CI: 1.10, 1.86, P=0.032), and no postoperative chemotherapy (HR: 1.54, 95%CI: 1.32, 1.70, P<0.001) were also significantly associated with poor DFS. The association between high MOR expression and DFS remained no statistically significant (HR: 1.19, 95%CI: 0.94, 1.43, P=0.167, **Table 3**). *OPRM1* analysis showed similar results (P=0.15, **Figure 3D**).

### Secondary Outcomes

The mean intraoperative sufentanil consumption was significantly higher in patients with high levels of MOR in their tumours (65.34 ± 4.80μg) than in those with cancers expression low levels of the receptor (45.60 ± 4.60μg) (P<0.001, **Figure 1A**). In terms of hospital LOS, the median duration 16.2 (14.6, 19.2) days in patients with high expression of MOR in their cancers, whereas the median LOS of those in the low MOR expression group was 15.8 (14.8, 18.7) days (P=0.597, **Figure 1B**).

Since PDACs are known to invade nerves and cause significant pain, we investigated the association between MOR expression and perineural invasion (**Figure 2B**). Interestingly, we found that a high expression of MOR was associated with higher rates of perineural invasion (72.9% *versus* 55.1%, P=0.036, **Figure 2B**). We also evaluated the association between survival and MOR expression in relation to opioids consumption (**Figures 1C, D**). Compared to the high MOR expression and high opioids (HMHO) consumption group, the OS of patients with low MOR expression in their tumours and low opioids (LMLO) consumption group was significantly longer (P=0.046, **Figure 1C**). Similarly, compared to the HMHO group, DFS in the LMLO group was significantly better than HMHO group (P=0.039, **Figure 1D**).

## Discussion

In this study, we aimed to investigate the impact of MOR and *OPRM1* gene expression on OS and DFS in PDAC patients who were candidate for curative surgery. The multivariate analysis indicated that high levels of MOR or *OPRM1* expression in PDAC tumours were not associated with worse OS and DFS. Our results are in agreement with findings recently reported by Steele at al. in 103 patients with advanced PDAC (11). Also, Oscar Díaz-Cambronero et al. found that there was no association between high expression and lower 5-year OS and DFS in colorectal cancers (10). In a cohort of 239 patients with esophageal squamous cell carcinoma, the expression of MOR did not affect survival (14). Contrarily, other studies demonstrated that high levels of MOR expression were a marker of worse prognosis in patients with laryngeal and lung cancers (8, 15).

A possible mechanism of opioid-induced cancer progression is via activation MOR and include angiogenesis, tumor-induced inflammation, and facilitation of epithelial-mesenchymal transition (5). Hence, we investigated the association between opioids consumption, MOR expression and long-term survival in cancer patients. Interestingly, we observed that patients requiring high intraoperative dosages of sufentanil and who also had PDAC tumours expressing high levels of MOR showed worse survival. This finding suggests an interaction between opioid use and MOR expression. Steele et al. reported that patients with metastatic PDAC requiring low dosages (oral morphine equivalents < 5 mg/day) of opioids had a significant longer survival than those being treated with high amounts (oral morphine equivalents ≥ 5 mg/day) of opioids (11). Of note, in that study patients in the high opioid group showed slightly higher expression of MOR (11). In a retrospective study, Zylla et al. demonstrated that high levels of MOR were associated with large opioid requirements in patients with advanced prostate cancers (16), also suggesting an interaction between MOR expression and opioid use. The results from Steele et al. and Zylla et al. are similar to our findings indicating an association between sufentanil use and higher MOR expression. It is unclear why patients with higher use of opioids show an exaggerated expression of MOR. Under basal conditions, MOR is down-regulated during agonist stimulation by accelerated degradation as well as the reduced receptor biosynthesis (17). This phenomenon is agonist-concentration and time-dependent. However, we could speculate that a dysregulated MOR turnover in malignant cells as the result of an inflammatory tumor microenvironment could explain our findings in which sustain stimulation with an agonist (opioid) impairs receptor desensitization, internalization, and down-regulation (18). Opioid analgesics have been used to manage moderate to severe acute and chronic pain during and after surgery. They are generally safe; however, in certain patients they produce adverse effects such as respiratory depression and vomiting which should be carefully considered.

Our work also demonstrates that while there was no difference between tumour tissue and adjacent tissue on MOR expression, specimens with higher levels of expression had higher perineural invasion. This finding *per se* is novel. Perineural invasion is associated with pain and is a marker of poor prognosis in patients with PDAC (19, 20). It remains from our study unknown why patients with higher levels of MOR had more features of perineural invasion. But, the presence of MOR in the brain has been linked to opioid modulation of neuritogenesis (21). Thus, we can speculate that MOR activation as the result of endogenous or exogenous opioids can trigger the release of soluble factors (i.e., nerve growth factor) that promote neurogenesis within the pancreatic cancer microenvironment (22). In our study, it is unlikely that a short period of exposure to sufentanil would trigger neurogenesis; therefore, we can speculate that elevated concentrations of locally released endorphins in patients with pain could be responsible of a high rate of neuritogenesis and perineural invasion (23). Alternatively, synthetic opioid such as fentanyl could promote neurotigenesis by regulating BNIP3 *via* miR-145-5 (24). BNIP3 pathway has shown to be upregulated in perineural invasion (25). But, none of our patients were taking opioid preoperatively to support this last theory.

Although our study benefit from large samples of stage I-III PDAC patients, we recognized important limitations as follows. First, the retrospective analysis may be associated with bias that have impact on findings. Second, the low rate of events might have limited the statistical power. Third, the fact that only intraoperative opioid use was recorded limits further conclusions on how postoperative opioids might have affected PDAC progression. And last, we did not perform studies to investigate why MOR expression was higher in patients requiring higher dosages of sufentanil or the relationship between the receptor and perineural invasion.

In conclusion, MOR expression was not associated with OS or DFS in stage I-III pancreatic cancer patients. Our results suggested high MOR expression associated with perineural invasion. Further investigations are needed to evaluate whether blockade of MOR during perioperative period might benefits in pancreatic cancer patients.

## Data Availability Statement

The raw data supporting the conclusions of this article will be made available by the authors, without undue reservation.

## Ethics Statement

The studies involving human participants were reviewed and approved by Fudan University Shanghai Cancer center (Protocol#2020106-1). The patients/participants provided their written informed consent to participate in this study.

## Authors Contributions 

Study design: HZ, JC, WC, and CM. Coordination: MDQ, ZS, and AG. Data acquisition: HZ, WC, and AG. Data interpretation: HZ, ZS, AG, and YY. Drafting: HZ, WC, and JC. All authors contributed to the article and approved the submitted version.

## Funding

This research was supported by the National Key Research and Development Program of China (NO. 2020YFC2008400), the National Natural Science Foundation of China (NO.81873948, 81871591,81801376), Clinical Research Plan of SHDC (NO. SHDC2020CR4064, SHDC2020CR1005A), Shanghai Shenkang hospital development centre clinical science and technology innovation project (NO. SHDC12018105), the Key Technology and Development Program of Shanghai (NO.17411963400); 2019 Fudan University Zhuo-Xue Project (JIF159607); Shanghai Leading Talent (NO: 2019-112); Shanghai Sailing program (21YF1406800); Natural Science Foundation of Shanghai (21ZR1413400).

## Conflict of Interest

The authors declare that the research was conducted in the absence of any commercial or financial relationships that could be construed as a potential conflict of interest.

## Supplementary Material

Supplementary Figure 1 Flowchart detailing the selection process for patients included in this retrospective analysis. **(A)** Flow chart of stage I-III pancreatic patients enrolled in this study; **(B)** Flow chart of stage I-III pancreatic patients clinical and RNAseq data from cancer database.
