# Supplementary figures and images for: Association of Mu-Opioid Receptor(MOR) Expression and Opioids Requirement With Survival in Patients With Stage I-III Pancreatic Ductal Adenocarcinoma
Source: Front Oncol. 2021 Jun 18;11:686877. doi: 10.3389/fonc.2021.686877 (PMC8249918; doi:10.3389/fonc.2021.686877)

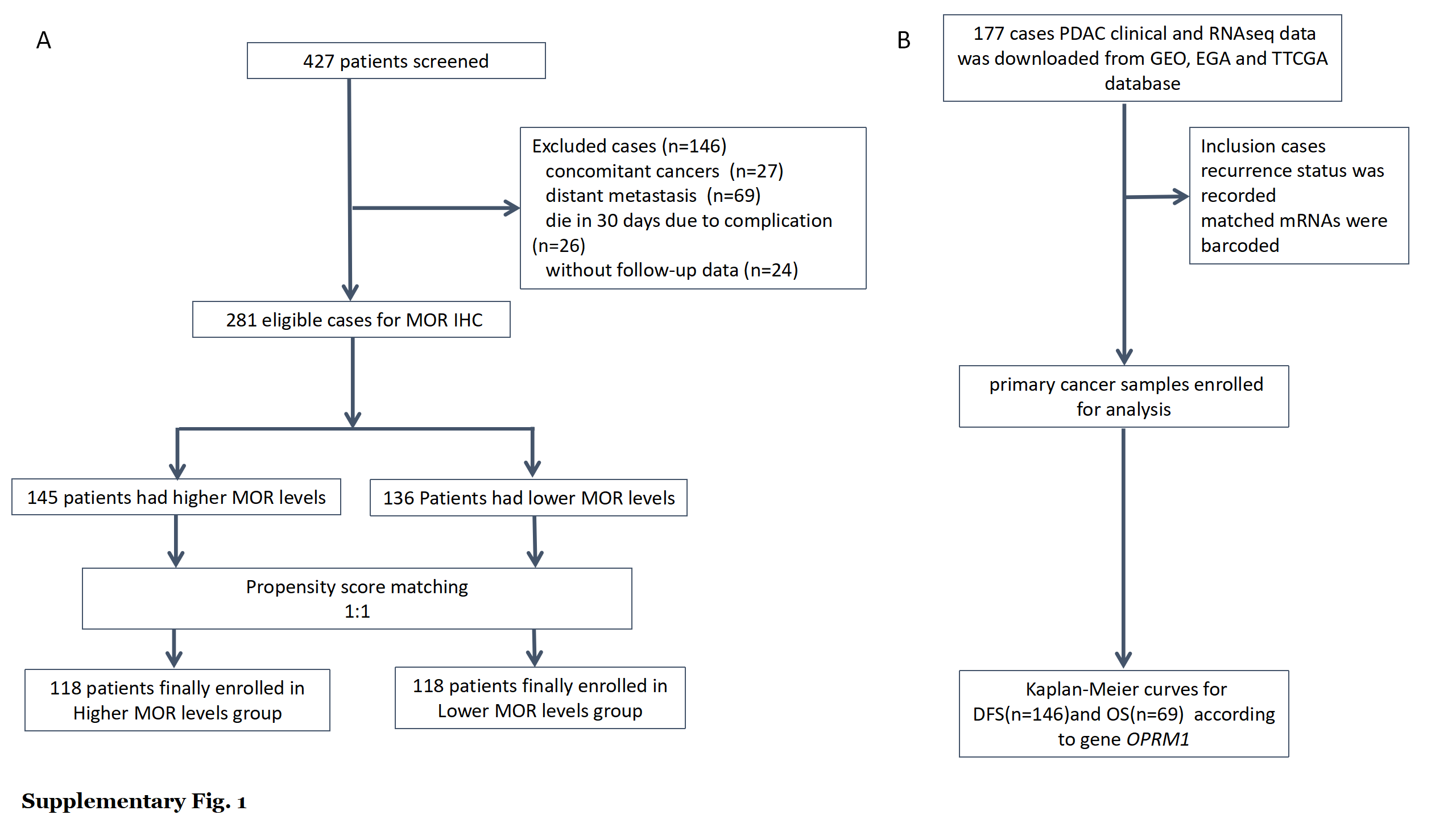

Supplement: Supplementary Figure 1 — Flowchart detailing the selection process for patients included in this retrospective analysis. (A) Flow chart of stage I-III pancreatic patients enrolled in this study; (B) Flow chart of stage I-III pancreatic patients clinical and RNAseq data from cancer database. [file Image_1.tif]
